# A Robust Design approach for error reduction in Thermoelastic Stress Analysis on Ti6Al4V alloy in presence of unknow biaxial residual stresses

**DOI:** 10.1016/j.heliyon.2025.e41780

**Published:** 2025-01-10

**Authors:** F. Di Carolo, U. Galietti

**Affiliations:** Department of Mechanics, Mathematics and Management, Polytechnic University of Bari, 70125, Bari, Italy

**Keywords:** Thermoelasticity, TSA general model, Robust design, Residual stress, Titanium alloy, Error reduction, Measurement error

## Abstract

In this study, a general model for Thermoelastic Stress Analysis (TSA) was employed in conjunction with simulated random noise sources to evaluate errors in the experimental technique using a robust design approach as a preliminary analysis. This facilitated the identification of an optimal experimental setup and anticipated ranges of measurement errors for TSA reducing testing time and errors. The model's validity was confirmed through TSA experiments conducted on AA2024 samples exhibiting biaxial residual stresses, as measured by a standard testing method. ANOVA (Analysis of Variance) and ANOM (Analysis of Means) analyses were conducted to explore the impact of parameters describing the analytical relationship between thermoelastic response and stresses in the presence of noise factors. The Robust Design approach was applied to the TSA measurement methodology, involving simulations of various noise sources and potential errors in the process, along with the application of different calibration methods.

## Highlights

• A general model for TSA was used to assess measurement errors through simulated random noise sources.
• A robust design approach was applied to identify an optimal experimental setup for TSA.
• ANOVA and ANOM were employed to assess the influence of parameters on the thermoelastic response under noisy conditions.
• The Robust Design approach involved simulations of various noise sources and the use of different calibration methods.
• The results offer error ranges and operational guidelines for applying the TSA technique in stress measurement.

**Table:** List of symbols

$\bar{\sigma }\;\left[MPa\right]$	Stress vector in a point
$\bar{\sigma_{M}}\;\left[MPa\right]$	Total mean stress
$\bar{\sigma_{m}}\;\left[MPa\right]$	Applied mean stress
$\Delta \bar{\sigma }\;\left[MPa\right]$	Amplitude of the applied stress
$\bar{\sigma_{r}}$	Residual stress vector
1, 2, 3	Reference system of the equation
1′,2′,3′	Principal reference system
θ	angle between the residual stress principal system and the reference system
ϕ	angle between the applied stress principal system and the reference system
$\overset{═}{R}$	Rotation matrix
$\gamma_{l}=\frac{{\Delta \sigma }_{2}}{{\Delta \sigma }_{1}}$	Ratio between the stress amplitude principal components
$\gamma_{r}=\frac{\sigma_{r2}}{\sigma_{r1}}$	Ratio between the residual stress principal components
t[s]	Time
$\frac{\omega }{2\pi }\;\left[Hz\right]$	Frequency
$\bar{\alpha }\;\left[K^{-1}\right]$	Vector of linear thermal expansion coefficients
$\rho \;\left[kg/m^{3}\right]$	Density
$C_{\varepsilon }\left[J/kg\;K\right]$	Specific heat at constant strain
$\bar{\bar{C}}\;\left[GPa\right]$	Stiffness matrix
$T_{0}\;\left[K\right]$	Reference temperature
ΔT[K]	Thermoelastic temperature variation
${\Delta T}_{nominal}$	Nominal thermoelastic temperature variation, evaluated from the TSA general model implementing the imposed value of applied stress, residual stress and material characteristics
${\Delta T}_{measured}$	Thermoelastic temperature variation measured by the simulated sensor
$k=\frac{\sigma_{mi}}{\Delta \sigma_{i}}$	ratio between the applied mean stress and the amplitude components (i = 1,2)
$\left[\Delta \sigma_{1}\;\gamma_{l}\;\phi \right]$	Characteristics tern identifying the set up
Y	Response of the studied system
$Y_{0}$	Ideal response of the studied system
SF, NF, and CF	Signal Factors, Noise Factors, and Control Factors
Q	Quality loss function
$K_{q}$	Economic loss
n	Number of samples
*μ*	Mean of Response
$S^{2}$	Variance of Response
*SN*	Signal to Noise ratio
TC Noise	Thermal Camera Noise

## 1 Introduction

The capability to measure stress and strain in a reliable and not invasive way is an important support in the Design of mechanical components and structures undergoing both static and dynamical load.

The Thermoelastic Stress Analysis (TSA) is a contactless technique capable to determine the superficial stress of a component exploiting the thermoelastic effect, which involves the generation of small reversible temperature variations, due to the volume change, in a component subjected to a dynamic load in a linear elastic field [1,2].

In recent years, the development of high performing IR cameras allowed the large employment of the TSA in several applications. In particular, the advantages of being contactless, full field, totally safe for the component and not requiring substantial surface preparation make the TSA a valid tool for testing real components and validating finite element models (FEM) [[3], [4], [5]].

The applicability of such a technique has been demonstrated in stress measurement [3,6,7], residual stress measurement [[8], [9], [10]], fracture mechanics [5,[11], [12], [13]] and fatigue characterization [[14], [15], [16]]. Moreover, TSA deployment as a stress measurement technique is consolidated and researcher validated calibration procedures aiming to obtain both a precise and accurate results [[17], [18], [19]].

Negligible variation of the mechanical characteristics with the temperature determines a proportional relation between the sum of the amplitude of the principal stresses and thermoelastic response. Despite most steels follow this behaviour, this approximation leads to a non-negligible error in stress measurement for materials such as aluminium and titanium alloys [8,9,20,21].

Lightweight titanium alloys are of great interest for applications in the aerospace and aeronautical sectors, where there is a need to reduce the weight-to-strength ratio. In these applications, structural components with more or less complex geometries can be subjected to loading systems that result in complex stress states. In such cases, the ability to identify the stress state non-destructively and over the entire field can be advantageous, both for providing input to numerical simulations and for component inspection during quality control.

In the works of Palumbo et al. [17] and Galietti et al. [9] a calibration correction has been validated, taking into account the mean load effect. The proposed procedure is based on Wong's second order equation [21] and it has been validated only in the case of isotropic materials subjected to monoaxial sinusoidal loads and monoaxial mean loads. Thus, the procedure cannot be applied to real components with complex geometry and generally oriented residual stress.

The general model proposed by Di Carolo et al. [10] allows to evaluate the thermoelastic response in the case of homogeneous and non-isotropic materials undergoing any loading conditions and residual stresses. In the works of Di Carolo et al. [20] and Palumbo et al. [22,23], the model validity was demonstrated. Di Carolo et al. carried out TSA experiments on AA2024 samples exhibiting biaxial residual stresses, as measured by a standard testing method, while in the work of Palumbo et al. the general model was combined with Westergaard's equations and Williams's series expansion to evaluate the SIF in pure titanium CT samples. The new formulation proved its validity when experimental results were compared with values from the ASTM standard formulas.

The presence of unknown residual stresses generally oriented usually biaxial in the real component is a big source of signal variation due to the sensitivity of the material to “mean stress effect” on thermoelastic effect leading to very significant error actually not assessed.

Aim of the study is to provide the best setup for TSA applied on Titanium alloys with unknown residual stresses using a robust design approach on simulated data obtained by the TSA General Model proposed by Di Carolo et al. [10] in the work. All the studies present in literature, including the revised higher order theory, are based on several assumptions that limit the application of such theory to the cases of isotropic materials subjected to uniaxial residual stresses and uniaxial applied loads. The novelty of the general model is its capability to evaluate the thermoelastic response in the case of homogeneous and non-isotropic materials undergoing any loading conditions and residual stresses.

The obtained setup will result as the less influenced by noise factors involved in the process and will provide more statistically robust output of the measurement campaign.

Despite the availability of an analytical model, the complexity of relations and the effect of random noise variables make the implementation of statistical methods a useful tool to study the applicability of the technique. This work performs a Robust Design like approach [24,25] of the TSA measurement system by applying statistic on an analytical model simulating also the various types of errors that can be made on the process.

This work in organised in two main phases. The first part is focused on the thermal signal; an ANOVA (Analysis of Variance) and ANOM (Analysis of Means) were performed to study the effect of the parameters describing the analytical relation between thermoelastic response and stresses in the presence of noise factors. The second part was performed implementing a Robust Design approach to the stress measurement system. The main results are presented in terms of the effect of the various process parameters on the statistics of the measurement error and its range.

## 2 Theory

The thermoelastic effect is based on the generation of small temperature variations associated with volume variations in materials subjected to loads in the linear elastic field.

The relation between temperature variations and applied load depends on the material characteristics and the loading system and is exploited to characterize the stresses distribution in components and structures by applying the TSA [2,6,7].

In adiabatic conditions, the relation between temperature variation and strains is [1]:

(1) $$\delta T=-\frac{3T\alpha K\delta \varepsilon }{\rho Cv}$$

Local adiabatic conditions can be obtained experimentally through the application of a sinusoidal load with a frequency high enough to make the heat transfer (conduction in the specimen or convection and radiance to the environment) neglectable. Thus, in the case of sinusoidal load and neglecting the dependence of the material characteristics on the temperature the thermoelastic response is proportional to the sum of the first scalar invariant [2]. In the case of a homogeneous and isotropic material, the TSA equation is:

(2) $$\frac{\Delta T}{T_{0}}=K_{0}\cdot \left(\Delta \sigma_{11\;}+\Delta \sigma_{22}\right)$$

ΔT in Eq. (2) represents the temperature variation generated by the application of the loading system with principal stress sum $\Delta \sigma_{11\;}+\Delta \sigma_{22}$ at the same frequency. *T*_*0*_ is the reference temperature and *K*_*0*_ is the thermoelastic parameter; for an isotropic material it depends on the material thermal expansion coefficient *α*, specific heat at constant pressure *C*_*p*_ and density *ρ* through the relation [2]:

(3) $$K_{0}=-\frac{\alpha }{\rho \cdot C_{p}}$$

For some materials such as aluminium and titanium alloys, the variations of the mechanical characteristics with temperature are not neglectable [20]. In this case the thermoelastic response is related to the loading characteristics through a more complex relation including second order effects. In the case of sinusoidal load for a homogeneous and generally anisotropic material in adiabatic conditions, the general thermoelastic equation can be written as follow [10]:

(4) $$\rho c_{\varepsilon }T^{-1}\Delta T=\left[{\left({\bar{\bar{C}}}^{-1}\left({\bar{\sigma }}_{m}+\bar{\bar{R}}{\bar{\sigma }}_{r}\right)\right)}^{T}{{\frac{\partial \bar{\bar{C}}}{\partial T}}^{T}|}_{\bar{\varepsilon }}-{\bar{\alpha }}^{T}{\bar{\bar{C}}}^{T}\right]{\bar{\bar{C}}}^{-1}\Delta \bar{\sigma }\sin\left(\omega t\right)+\frac{1}{4}\left[{\left({\bar{\bar{C}}}^{-1}\Delta \bar{\sigma }\right)}^{T}{{\frac{\partial \bar{\bar{C}}}{\partial T}}^{T}|}_{\bar{\varepsilon }}\right]{\bar{\bar{C}}}^{-1}\Delta \bar{\sigma }\left[1-\cos\left(2\omega t\right)\right]$$

In Eq (4).

- ΔT is the temperature difference due to the thermoelastic effect associated with the stress $\Delta \bar{\sigma }\sin\left(\omega t\right)+{\bar{\sigma }}_{m}$;
- $\bar{\varepsilon }$, $\bar{\;\alpha }$ and $\bar{\bar{C}}$ represent respectively the state of deformation in a point, the vector of the linear thermal expansion coefficients and the material stiffness matrix. All can be expressed in a generic system of axes *x y* and *z*;
- ${\bar{\sigma }}_{r}$ is the residual stress vector expressed in the principal system;
- the tensor $\bar{R}$ is the rotation matrix that allows writing ${\bar{\sigma }}_{r}$ in the reference system used to write the equation and depends on the angle *θ* between the residual stress principal system and the reference system.

Typical TSA analysis is conducted with a lock-in amplifier, allowing to discriminate the amplitude of the signal at the same frequency of the load.

By focusing on the semi-amplitude of the temperature running at the same frequency as the applied load, one can describe the temperature variations as:

(5) $$\Delta T={\left(\rho C_{\varepsilon }\right)}^{-1}T_{0}\left[{\left({\bar{\bar{C}}}^{-1}\left({\bar{\sigma }}_{m}+\bar{\bar{R}}{\bar{\sigma }}_{r}\right)\right)}^{T}{\frac{\partial \bar{\bar{C}}}{\partial T}}^{T}-{\bar{\alpha }}^{T}{\bar{\bar{C}}}^{T}\right]{\bar{\bar{C}}}^{-1}\Delta \bar{\sigma }$$

In the plane it becomes:

(6) $$\rho c_{\varepsilon }{T_{0}}^{-1}\Delta T=\left[{\left({\left(\begin{array}{ccc} C_{1111} & C_{1122} & C_{1112}\\ C_{2211} & C_{2222} & C_{2212}\\ C_{1211} & C_{1221} & C_{1212} \end{array}\right)}^{-1}\left(\left(\begin{array}{c} \sigma_{mxx}\\ \sigma_{myy}\\ \sigma_{myx} \end{array}\right)+\left[\begin{array}{ccc} \cos^{2}\;\theta & \sin^{2}\;\theta & 2\;\cos\;\theta \;\sin\;\theta \\ \sin^{2}\;\theta & \cos^{2}\;\theta & -2\;\cos\;\theta \;\sin\;\theta \\ -\cos\;\theta \;\sin\;\theta & \cos\;\theta \;\sin\;\theta & \cos^{2}\;\theta -\sin^{2}\;\theta \end{array}\right]\left(\begin{array}{c} \sigma_{r11}\\ \sigma_{r22}\\ 0 \end{array}\right)\right)\right)}^{T}{\left(\begin{array}{ccc} \frac{{\partial C}_{1111}}{\partial T} & \frac{{\partial C}_{1122}}{\partial T} & \frac{{\partial C}_{1112}}{\partial T}\\ \frac{{\partial C}_{2211}}{\partial T} & \frac{{\partial C}_{2222}}{\partial T} & \frac{{\partial C}_{2212}}{\partial T}\\ \frac{{\partial C}_{1211}}{\partial T} & \frac{{\partial C}_{1221}}{\partial T} & \frac{{\partial C}_{1212}}{\partial T} \end{array}\right)}^{T}-{\left(\begin{array}{c} \alpha_{xx}\\ \alpha_{yy}\\ \alpha_{yx} \end{array}\right)}^{T}{\left(\begin{array}{ccc} C_{1111} & C_{1122} & C_{1112}\\ C_{2211} & C_{2222} & C_{2212}\\ C_{1211} & C_{1221} & C_{1212} \end{array}\right)}^{T}\right]{\left(\begin{array}{ccc} C_{1111} & C_{1122} & C_{1112}\\ C_{2211} & C_{2222} & C_{2212}\\ C_{1211} & C_{1221} & C_{1212} \end{array}\right)}^{-1}\left(\begin{array}{c} {\Delta \sigma }_{xx}\\ \Delta \sigma_{yy}\\ \Delta \sigma_{yx} \end{array}\right)$$

Eq. (6) can be used to evaluate the effect of residual stresses on the thermoelastic response of materials with known mechanical and physical characteristics.

**Fig. 1:**
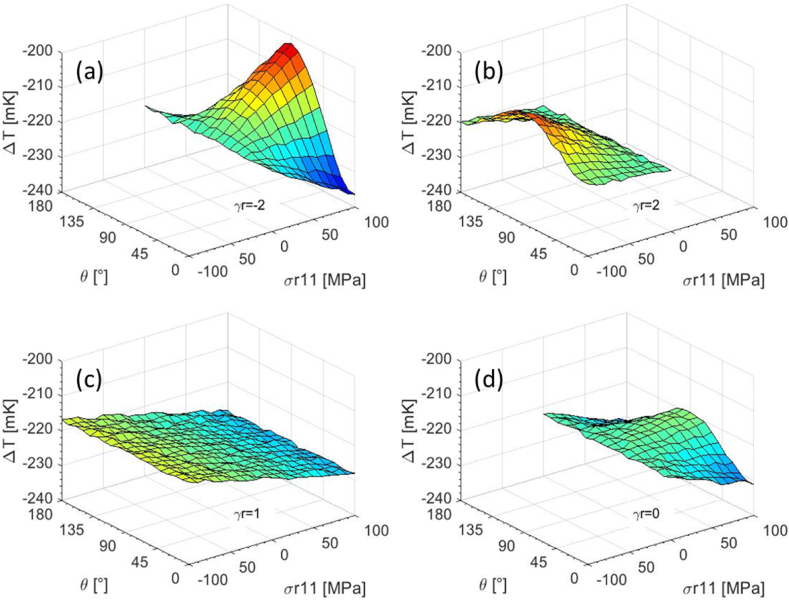
Thermoelastic signal as a function of the two independent variables *σ*_*r11*_ and *θ*: (a) γ_r_ = −2, (b) γ_r_ = 2, (c) γ_r_ = 1 (d) γ_r_ = 0 [10].

The surfaces in Fig. 1 show the thermoelastic behaviour of the alloy Ti6Al4V as a function of the residual stress characteristics ($\sigma_{r11}$, *θ* and the ratio $\gamma_{r}=\sigma_{r22}\;/\sigma_{r11}$). The effect of residual stress is higher when the principal system is tilde of 90° and the two principal components are opposite in sign (i.e. traction-compression).

All the surfaces in Fig. 1 have been obtained imposing same material characteristics (nominal values in Table 1) and same loading conditions (monoaxial sinusoidal load with *Δσ* = 200 MPa and *σ*_*m*_ = 220 MPa); a white gaussian noise with a standard deviation of 0.01K, obtained experimentally [10], was also added.

**Table 1:** Values for material characteristics of both the traction and compression case simulation plan [[28], [29], [30], [31], [32]].

	***Nominal value***	***val1***	***val2***
***α [1/K]*** [[28], [29], [30],^32^]	8.9E-6	8.6E-6	9.1E-6
***E [GPa]*** [[28], [29], [30],^32^]	115	110	119
***ρ [Kg/m***^***3***^***]*** [[28], [29], [30],^32^]	4.471E3	4.429E3	4.512E3
***C***_***ε***_***[J/Kg K]*** [[28], [29], [30],^32^]	552	533	570
∂E/∂T***[MPa/K]*** [31]	−48	−46	−50

## 3 Methodology

The analytical solution expressed in Eq. (6) enables the simulation of thermoelastic response once the material characteristics, loading conditions and residual stresses are known. However, the accuracy of TSA in assessing stresses and residual stresses is susceptible to various sources of error. These errors are challenging to isolate and compensate due to the intricate interplay between dependent parameters.

In this context, a statistical approach, applied through the test simulation analysis, can help to identify the most influential parameters or group of parameters and their effects, with the aim of optimizing and ensuring a robust measurement technique.

A physical model which behaviour is described by an equation with many variables, such as, for instance, the temperature variation due to thermoelasticity described by TSA general model, can be used in many different approaches according to the aim of the work. For example, stresses can be determined with TSA general model measuring temperature variation, but in this case, unknown residual stresses must be treated as “noise” factors since they will provide a measurement variation and then an error. The same general model can be used to assess the unknown residual stresses as a function of temperature variation, in this case the characteristics of material variability must be treated as noise factor since it will provide a signal variability [26].

In the case of presence of residual stresses in TSA, the variability of the measurement given by noise factors (factors that cannot be controlled) can overwhelm the signal variability due to the factors which influence we want to assess and analyse of. In this case, a statistical approach that provide both statistical based models and statistical significance of each factors can help in both assess the overall error due to noise factors and provide info of the optimal setup for the best signal to noise ratio.

In this study, the analytical model (Eq. (6)) was utilized to conduct full factorial simulations to evaluate TSA performance. Initial ANOVA and ANOM were performed to identify the primary parameters and interactions affecting the measurement. The results were then compared with the analytical description to distinguish between effects covered by noise factors and those involving significant variations that can be effectively recorded.

Subsequently, the Robust Design method was employed to optimize the measurement performance in stress measurement by comparing three different calibration methods.

Table 2 lists the analyses conducted and reports parameter classifications. The noise sources considered in the study encompassed the IR camera noise (TC Noise), the residual stress tensor (defined by the principal stress components and their orientation), and the material characteristics variability in respect to nominal values.

**Table 2:** Analysis performed and parameters classification.

***Analysis***	***Input***	***Noise***	***Output***
***ANOVA and ANOM***	• Material characteristics • Residual stress vector	• TC Noise	• p-values • Means
***Parameters Robust Design***	• $\Delta \bar{\sigma }$	• TC Noise • $\bar{\bar{R}}{\bar{\sigma }}_{r}$ • Material characteristics variation	• Δσmeas • SN • Q

The parameter design endeavours to minimize performance sensitivity to various sources of variation while ensuring minimal quality loss in the response. Key indicators include the *Signal-to-Noise ratio* (*SN*) and the *Quality Loss Function* (*Q*) [25,27]. In the conventional application of Robust Design, the quality loss function denotes the cost incurred once the product is operational. However, in this study, it has been normalized with respect to the costs (*K*) and signifies the deviation of the response from the correct value.

## 4 Effect of the main sources of error on the TSA measurement

This paragraph presents the analysis carried out to identify the main variables that cause errors in measuring stresses using the thermoelastic technique. The tools used are ANOVA and ANOM, as merely observing the analytical model cannot determine whether signal variations caused by changes in certain variables or interactions between variables are measurable and significant, rather than being covered by noise.

### 4.1 Material

The study was conducted by taking into account the physical and mechanical properties of the Ti6Al4V alloy, wherein its thermoelastic behaviour is influenced by a second-order effect [20]. The material properties and their range of variability were determined based on values found in the literature [[28], [29], [30], [31], [32]]. The amplitude and average load were chosen with consideration of the material's mechanical properties and yield strength to ensure linear elastic conditions. Residual stress vectors were determined by referencing both typical literature values and preliminary results obtained from an analytical study of the thermoelastic behaviour of the Ti6Al4V alloy. The study was carried out by implementing a simulation plan that describes the actual operational conditions encountered in TSA measurements.

### 4.2 Parameters description and statistical test plan based on simulations

As stated, the influence of primary parameters impacting thermoelastic response were investigated by conducting ANOVA and ANOM tests on data generated using an analytical model and simulated noise.

To replicate IR camera noise, a white Gaussian noise with a Standard Deviation of 0.01 K (determined experimentally for a typical camera with an integration time of 2500μs [10]) was added to the response calculated using Eq. (6), with 5 repetitions performed.

Eq. (6) delineates a complex relationship between load and residual stress, contingent upon the mutual configuration of the two tensors. Consequently, the same residual stress state may amplify or reduce the signal based on the applied load's orientation and sign.

In order to remark the effect of the grade of biaxiality of the load on TSA measurement, in this analysis the ratio among the principal stresses was used to identify the level of the *Δσ*_*22*_ as a function of *Δσ*_*11*_. Consequently, the simulation plan was split in two using one plan to describe the effect of tension-tension and tension-compression loads (with γ assuming respectively positive and negative values) and another plan dedicated to compressive-compressive loads that are characterized by higher values (being the elastic limit higher in compression) and only positive values for the ratio g being both values negative.

**Fig. 2:**
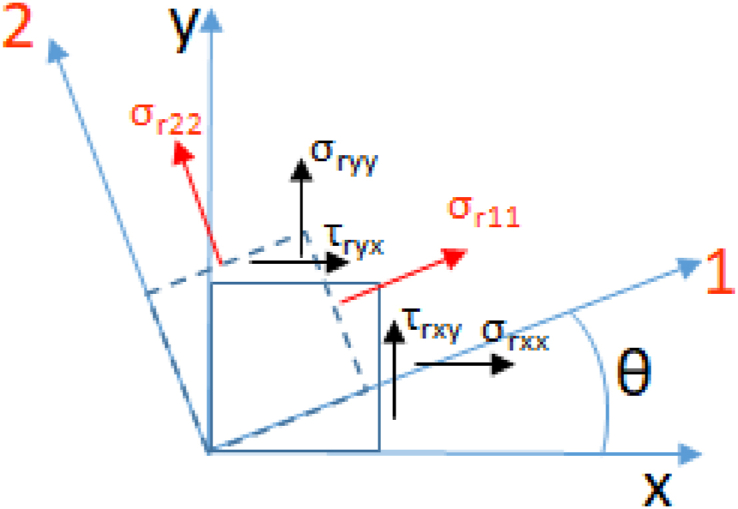
Residual stresses principal system and loading system.

Residual stress characteristics is defined by *σ*_*r11*,_ *σ*_*r22*_ and *θ* (Fig. 2). The plan was formulated to represent *σ*_*r22*_ via the difference between the principal components *σ*_*r11*_*-σ*_*r22*_ to get a simulation plan incorporating all conceivable residual stress configurations while ensuring *σ*_*r11*_*> σ*_*r22*_.

The two subplans (outlined in Table 3 and Table 4) were designed, considering all statistically relevant residual stress configurations.

- *Δσ*_*11*_: Three values for the first principal component of load amplitude for each subplan.
- *γ*: Three values for each subplan for the ratio between the principal components of load amplitude, simulating traction-traction and traction-compression conditions for subplan 1 and compression-compression conditions in subplan 2.
- *φ*: Three values for the angle between the principal loading system and the reference system.
- $k=\frac{\sigma_{m11}}{{\Delta \sigma }_{11}}=\frac{\sigma_{m22}}{{\Delta \sigma }_{22}}$: Three values for the ratio between the mean load and the load amplitude components, assumed constant in every direction of the amplitude tensor.
- *σ*_*r11*_: Seven different values for the first principal component of the residual stress tensor.
- *σ*_*r11*_*-σ*_*r22*_: Three values for the difference between the principal components of the residual stress system.
- *θ*: Three values for the angle between the principal residual stress system and the reference system.
- Two different values for each material characteristic (refer to Table 1).

**Table 3:** Values for the load and residual stress characteristics in the simulation subplan 1.

	***lev1***	***lev2***	***lev3***	***lev4***	***lev5***	***lev6***	***lev7***
***Δσ***_***11***_***[MPa]***	100	150	200	–	–	–	–
***γ***_***l***_	−0.5	0	1	–	–	–	–
***φ***	0	45	90	–	–	–	–
***k***	0	1	1.5	–	–	–	–
***σ***_***r11***_***[MPa]***	−150	−100	−50	0	50	100	150
***σ***_***r11***_***-****σ*_***r22***_***[MPa]***	0	100	150	–	–	–	–
***θ***	45	90	–	–	–	–	–

**Table 4:** Values for the load and residual stress characteristics in the simulation subplan 2.

	***lev1***	***lev2***	***lev3***	***lev4***	***lev5***	***lev6***	***lev7***
**Δσ**_**11**_**[MPa]**	−100	−200	−300	–	–	–	–
**γ**_**l**_	1	1.5	2	–	–	–	–
**φ**	0	45	90	–	–	–	–
**k**	0	1	1.5	–	–	–	–
**σ**_**r11**_**[MPa]**	−150	−100	−50	0	50	100	150
**σ**_**r11**_**-σ**_**r22**_**[MPa]**	0	100	150	–	–	–	–
**θ**	45	90	–	–	–	–	–

### 4.3 ANOVA and ANOM simulation workflow

**Fig. 3:**
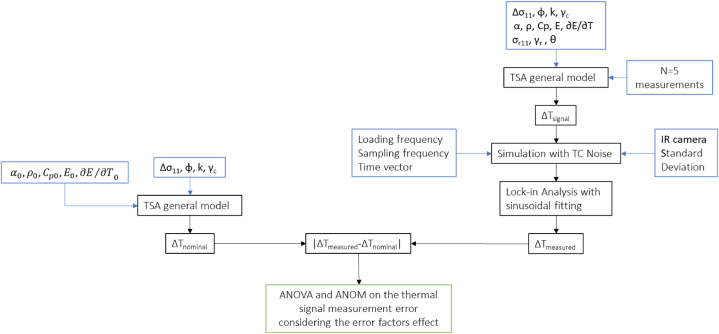
Workflow for the ANOVA and ANOM

Fig. 3 illustrates the flow chart detailing the simulation steps for generating data for the ANOVA.

The nominal TSA value ${\Delta T}_{nominal}$ represents the amplitude of the thermal signal calculated using Eq. (6) with nominal values for material characteristics (refer to Table 1), disregarding the presence of residual stress value expected. This value is used as a reference value.

For each combination of the noise parameters, *ΔT*_*signal*_ was calculated by using Eq. (6) and a synthetic data vector was generated with a loading frequency of 17Hz and a time vector of 10 s, sampled at 200 Hz [10]. Additionally, white Gaussian noise was added. Subsequently, a lock-in analysis on simulated data was conducted to derive *ΔT*_*measured*._ The lock-in algorithm is based on the periodic signal reconstruction through a least square fitting with known frequency of the signal [Riferimento ad uno dei nostril paper]. The observed response was then determined as the relative error:

(9) $$Er=\left[\left({\Delta T}_{nominal}-{\Delta T}_{measured}\right)/{\Delta T}_{nominal}\right]\cdot 100$$

The ANOVA results indicated which among the significant variables influence the response considered, namely the error in measuring the thermal signal. The subsequent application of ANOM guided us in understanding which parameters most significantly affect this response [32].

### 4.4 ANOVA and ANOM results and discussion

It is now possible to assess which of the parameters that, according to TSA general model, are supposed to influence the TSA measurement are actually significant. To be significant a parameter has to provide a signal that overcome, statistically speaking, the inherent noise of the measurement.

Table 5 presents only the results, expressed in p-values, for subplan 1 related to the individual factors and the significant first-order interactions, while the complete results for subplans 1 and 2 are provided in Appendix 1.

**Table 5:** ANOVA results for simulation subplan 1.

*Source*	*Sum Sq.*	*d.f.*	*Mean Sq.*	*F*	*Prob > F*	Note (a.b)**
***α***	5.7E+06	1	5.7E+06	5.3E+05	0.0E+00	a
***ρ***	6.9E+05	1	6.9E+05	6.4E+04	0.0E+00	a
***C***_***ε***_	9.0E+06	1	9.0E+06	8.3E+05	0.0E+00	a
***E***	1.4E+05	1	1.4E+05	1.3E+04	0.0E+00	a
***∂E/∂T***	4.0E+04	1	4.0E+04	3.7E+03	0.0E+00	a
***σ***_***r11***_	5.6E+06	6	9.3E+05	8.6E+04	0.0E+00	a
***Δσ***_***rp***_	4.0E+05	2	2.0E+05	1.8E+04	0.0E+00	a
***θ***	4.2E+04	2	2.1E+04	1.9E+03	0.0E+00	a
α *ρ	5.0E+02	1	5.0E+02	4.6E+01	1.2E-11	a
α * C_***ε***_	6.5E+03	1	6.5E+03	6.0E+02	2.1E-132	a
ρ * C_***ε***_	7.7E+02	1	7.7E+02	7.1E+01	3.0E-17	a
ρ * σ_***r11***_	4.8E+02	6	8.0E+01	7.3E+00	7.4E-08	a
***C***_ε_*E	1.6E+02	1	1.6E+02	1.5E+01	1.2E-04	a
***C***_ε_*∂E/∂T	4.8E+01	1	4.8E+01	4.5E+00	3.5E-02	b
***C***_ε_* σ_***r11***_	6.3E+03	6	1.0E+03	9.6E+01	3.1E-121	a
***C***_ε_* Δσ_***rp***_	4.2E+02	2	2.1E+02	2.0E+01	3.1E-09	a
E*∂E/∂T	2.5E+02	1	2.5E+02	2.3E+01	1.3E-06	a
E* σ_***r11***_	3.4E+04	6	5.7E+03	5.2E+02	0.0E+00	a
E* Δσ_***rp***_	2.4E+03	2	1.2E+03	1.1E+02	1.4E-48	a
E* θ	2.6E+02	2	1.3E+02	1.2E+01	6.4E-06	a
∂E/∂T * σ_***r11***_	9.6E+03	6	1.6E+03	1.5E+02	6.8E-188	a
∂E/∂T * Δσ_***rp***_	6.7E+02	2	3.4E+02	3.1E+01	3.3E-14	a
***Δσ***_rp_*θ	2.3E+04	4	5.8E+03	5.4E+02	0.0E+00	a

**a as expected from the analytical model, *b* should be significative from the analytical model, but it results covered by noise.

In both cases, all material characteristics (*α, ρ, C*_*ε*_*, ∂E/∂T* and *E*) and all the three residual stress system characteristics (*σ*_*r11*_, Δ*σ*_*rp*_and *θ*) exhibit p-values lower than 0.05, affirming their significance on the relative error.

These results show the factors and the two-terms interactions whose effects are covered by sources of error, as evidenced by p-values exceeding 0.05 despite their indicated significance in the analytical model (as indicate in the 7th column of Table 5).

Many interactions are deemed non-significant, despite their direct connection to temperature variation in Eq. (6). The analytical model provides the exact relation between parameters and thermoelastic response but lacks information regarding their impact on the measured signal, which depends on all noise factors.

**Fig. 4:**
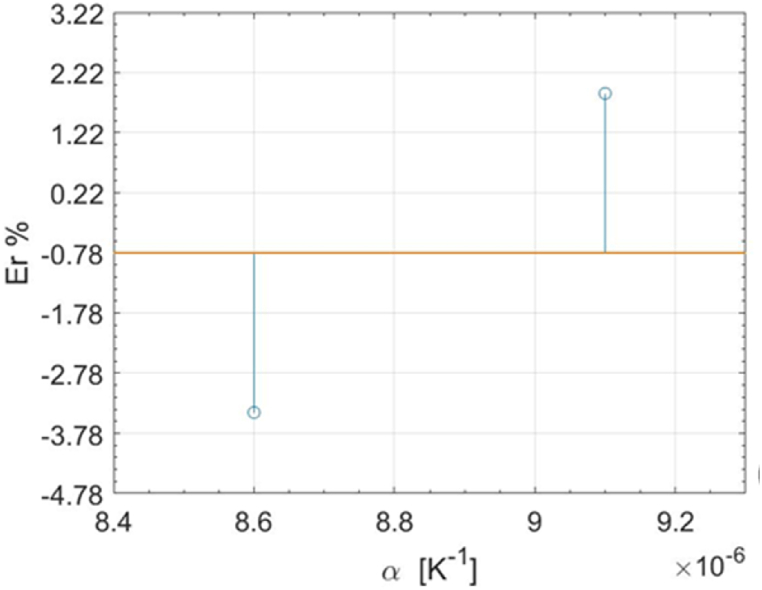
ANOM results for the simulation subplan 1. The Means are plotted for the different levels of the material characteristics *α.*

**Fig. 5:**
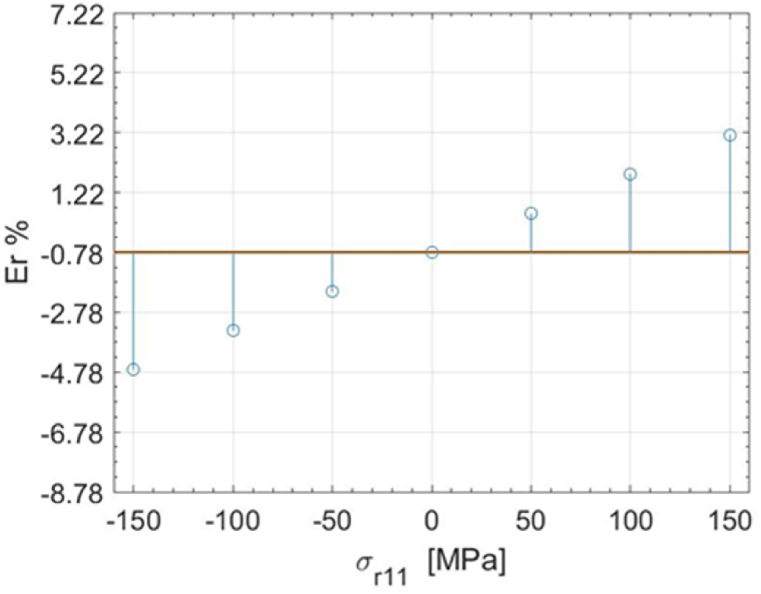
ANOM results for the simulation subplan 1. The Means (blue circles) are plotted for the different levels of the residual stress characteristics *σ*_*r11*_.

In Fig. 4, Fig. 5, as an example, the most relevant results of the ANOM are presented; the complete results are provided in Appendix 2. In Fig. 4 the plotted means for each level of the material characteristics *α* in the traction case are provided, while Fig. 5 shows the means obtained for each level of the residual stress characteristics *σ*_*r11*_. There is clear evidence of a significant difference in the mean of the TSA response across different levels of all material characteristics. Specifically, variations in *α* and *C*_*ε*_ lead to a higher signal variation, attributed both to their direct effect on the thermal signal and to the range selected for simulation. A less pronounced variation is instead attributed to the change in the mechanical properties of the material.

Similar trends are observed for the compression case. A comparison between the two cases highlights a higher overall error mean in the compression case, characterized by more critical loading conditions. Additionally, this condition induces a greater influence of the amplitude of residual stresses but a lesser effect of their orientation.

The results obtained show how the effect of each parameter is closely tied to the ranges chosen for both the error sources and the parameters themselves. Additionally, the results demonstrate that all the considered parameters are significant, and their variation statistically influences the error on the TSA measurement. This confirms the necessity of a statistical model including all these parameters to define the most robust operating conditions for measuring stresses using the TSA technique.

## 5 Robust design application: sum of stresses measurements methodology

This part of the study focused on addressing the issue of stress measurement using TSA. The objective was to determine the loading characteristics that would lead to more accurate and robust stress measurements by employing the classical TSA equation (Eq. (1)). This was accomplished by comparing three different calibration methods.

1. Estimation of the thermoelastic constant using nominal values for the material characteristics (calibration method 1).
2. Estimation of the thermoelastic constant through simulation of an experimental calibration using relaxed dog-bone samples of the same material (calibration method 2).
3. Estimation of the thermoelastic constant through simulation of an experimental calibration using dog-bone samples of the same material, under identical residual stress conditions but with variable orientations for the principal system of residual stress (Calibration method 3).

These calibration methods are all predicated on the proportional relationship between the thermoelastic response and the sum of the principal stress variation. Despite the existence of calibration procedures based on the second-order effect [6,7], they couldn't be compared in this study due to their limitation to cases of uniaxial loading and uniaxial residual stress.

### 5.1 Sum of stresses measurements: problem definition and workflow

**Fig. 6:**
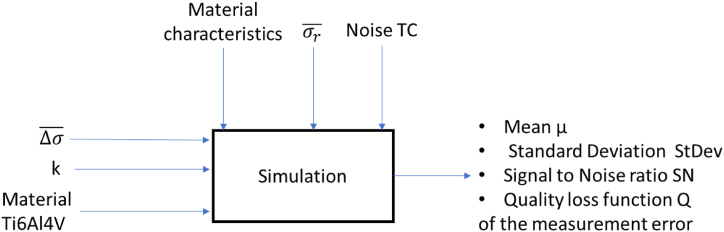
Block diagram of the TSA stress measurement system.

Fig. 6 shows the block diagram representation of the system. In the context of TSA application for stress measurement, the control factors are the loading characteristics, while the noise factors include variations in material characteristics, residual stress system characteristics, and IR camera noise.

The study utilized the Taguchi method, wherein a control factors matrix was constructed comprising three different values for each control factor. Additionally, an external matrix was developed, considering two values for the material characteristics, three values for the residual stress system characteristics, and three values for the IR camera noise.

The specific values considered for the IR camera noise were [27]: $\mu_{Noise}-\sqrt{3/2}{\cdot \sigma }_{Noise},\mu_{Noise}\;\text{and}\;\mu_{Noise}+\sqrt{3/2}{\cdot \sigma }_{Noise}$.

Where $\mu_{Noise}$ and ${\cdot \sigma }_{Noise}$ represent the mean value and variance of the noise affecting the amplitude of the thermal response measurement, obtained by simulating a high number of repetitions of the ΔT measurement with the lock-in analysis.

The values assumed for the control factor and the remaining noise factors are listed in Table 1, Table 3, Table 4. As there was no limit on the number of simulations, both the control factor matrix and the external matrix were designed as full factorial plans.

**Fig. 7:**
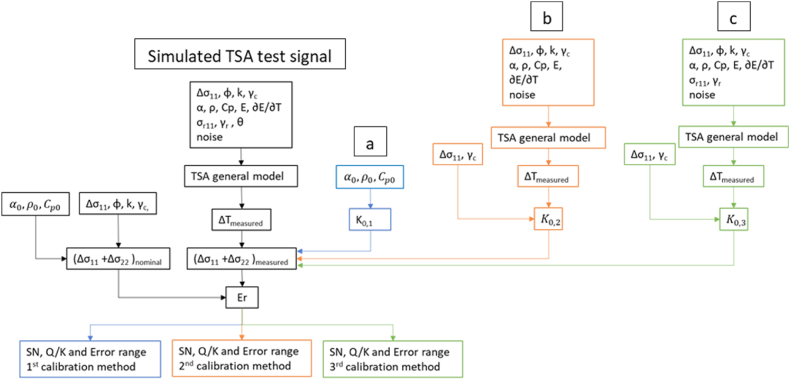
Workflow of the TSA stress measurement system Robust Design. (a) Calibration method 1, (b) Calibration method 2, (c) Calibration method 3. The error was evaluated for esch calibration method comparing the simulated nominal signal with the simulited measurement.

The steps involved in processing the simulation results are detailed in Fig. 7. The thermoelastic response, *ΔT*_*measured,*_ was utilized to evaluate the sum of the principal stress by employing the classical approach.

The first calibration method was applied calculating the thermoelastic constant as follows:

(11) $$K_{0}=\frac{\alpha_{0}}{\rho_{0}\cdot C_{p0}}$$

The second and third calibration methods were applied by simulating experimental calibrations. For each combination of control factors and each combination of error factor, the calibration constant *K*_*0*_ was determined by simulating the calibration procedure on a dog-bone sample while maintaining the same values of *k* and *Δσ*.

In the second calibration, the simulation was conducted using a virtual sample of the same material with no residual stresses, in the third kind of calibration the virtual sample is affected by non-null residual stress conditions, of about the same magnitude used in the analysis, but with variable orientations for the residual stresses principal reference system.

For all cases, the sum of principal stresses was evaluated as:

(12) $${\left({\Delta \sigma }_{11}+{\Delta \sigma }_{22}\right)}_{measured}={K_{0}}^{-1}\frac{{\Delta T}_{measured}}{T_{0}}$$

The stress measurement obtained using Eq. (13) was subsequently compared with the input-imposed value, and the relative error was evaluated as:

(13) $$Er=\frac{{\left({\Delta \sigma }_{11}+{\Delta \sigma }_{22}\right)}_{imposed}-{\left({\Delta \sigma }_{11}+{\Delta \sigma }_{22}\right)}_{measured}}{{\left({\Delta \sigma }_{11}+{\Delta \sigma }_{22}\right)}_{imposed}}\cdot 100$$

The value calculated with Eq. (13) serves as an indicator of the measurement's performance, and the optimization problem aims to minimize this value along with its variance.

The problem is of type “*smaller the batter”* and the *signal to noise ratio* and the *quality loss function* were computed for each value *j* of each control factor as follows [27]:

(14) $${SN}_{ij}=-10\cdot \log\left(\frac{1}{N_{ij}}\sum_{n=1}^{N_{ij}}{y_{n}}^{2}\right)$$

(15) $$\frac{Q_{ij}}{K}={X_{ij}}^{2}+{S_{ij}}^{2}$$

Where the subscript *ij* indicates the value j of the *i-th* control factor. *N*_*ij*_ is the number of combinations of control factors with control factor *i* having value *j* and *y*_*n*_ is the mean of all the values of *Er* for the *n*_*th*_ control factor combination.

It is crucial to note that in assessing stress measurement performance on real components, the only parameter adjustable by the operator is the ratio *k*, which represents the ratio between the mean and the amplitude of the load. This ratio remains constant for every pixel of the IR camera. In cases involving complex geometries or operational loads, the stress distribution can be quite complex, and the tension tensor cannot be treated as a control factor.

The statistical analysis provided the evaluation of the *SN* and *Q/K* (Eq. (14) and Eq. (15)) for each value of *k* in the plan. Once the optimal value of *k* was identified, the impact of the loading characteristics on measurement error was investigated by assessing the same indicators for each of their values, focusing solely on the tests of the plan with the optimal *k*.

Finally, for each calibration technique, the overall error range was determined as μ±3S, where μ and *S* represent the mean and standard deviation of the relative errors of the optimal-*k* simulation plan.

### 5.2 Sum of stresses measurements: results and discussions

#### 5.2.1 Effect of the k ratio

For each calibration method, the results are presented in terms of the error statistics associated with each value of the factor k both for the simulation subplan 1 (Table 6) and the simulation subplan 2 (Table 7). Although all calibration methods exhibit comparable values of standard deviation of the relative error, the third calibration method consistently demonstrates lower means of the relative error in every condition.

**Table 6:** simulation subplan 1. Means and Means of the Standard Deviations of the relative error for each value of the control factor *k* and each calibration method.

		*calib.1*		*calib.2*		*calib.3*	
		*Mean Error %*	*StDev Error %*	*Mean Error %*	*StDev Error %*	*Mean Error %*	*StDev Error %*
*k*	0	−0.9	6.5	−1.0	4.5	0.2	3.8
	1	8.3	6.7	−1.0	4.0	0.1	3.3
	1.5	12.9	7.0	−1.0	3.8	0.1	3.0

**Table 7:** simulation subplan 2. Means and Means of the Standard Deviations of the relative error for each value of the control factor k and each calibration method.

		*calib.1*		*calib.2*		*calib.3*	
		*Mean Error %*	*StDev Error %*	*Mean Error %*	*StDev Error %*	*Mean Error %*	*StDev Error %*
*k*	0	−1.1	5.5	−1.2	3.1	2E-03	0.5
	1	−9.2	5.5	−1.4	3.4	3E-03	0.6
	1.5	−13.2	5.5	−1.4	3.6	3E-03	0.6

**Fig. 8:**
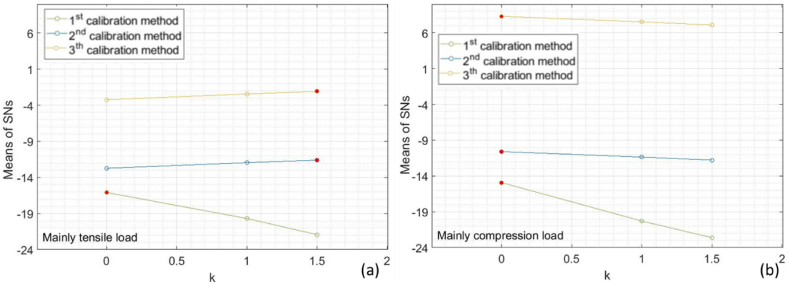
Means of *SN*s of the relative error for each value of the control factor *k* and each calibration method for (a) the simulation subplan 1 (b) the simulation subplan 2.

In the case of simulation subplan 1, the plots of the signal-to-noise ratio (*SN*) (Fig. 8) show how the quality of the measurement improves with the mean load only with the first calibration, while for the two experimental calibrations, the mean load mitigates the effect of error sources stemming from residual stress. This effect is more pronounced with the second calibration method, wherein the calibration sample is relaxed, and residual stress is completely neglected.

Similarly, in the case of simulation subplan 2, all three calibration methods exhibit a similar trend *Q/K*: a decrease of the quality of the measurement with the mean load.

The measurements under compressive load exhibited superior measurement performance obviously due to higher loads and then higher related signals.

In real stress measurement scenarios, controlling the direction of the load is not easy. Therefore, it is useful to provide only one optimal *k* value for a given calibration, regardless of the load direction. For the first calibration method, the optimal value is *k* = 0, which corresponds to a better *SN Q/K* for each load condition. For the second and third calibration methods the optimal operating solution is to adopt the highest possible *k* value, as it leads to a more significant improvement in performance under tensile loading compared to the increase that would occur with a null mean load in compression.

Consequently, in order to compare the best performance possible, the effect of the loading characteristics on the measurement error was investigated by evaluating the Means of *SN*s *Q/K* for each of their values, considering all the tests with *k* = 0 for the first calibration method and with *k* = 1.5 for the second and third calibration methods.

The results obtained for the *Q/K* ratio are consistent with those observed for the *SN* ratio; detailed results are provided in Appendix 3.

#### 5.2.2 Effect of the loading characteristics

**Fig. 9:**
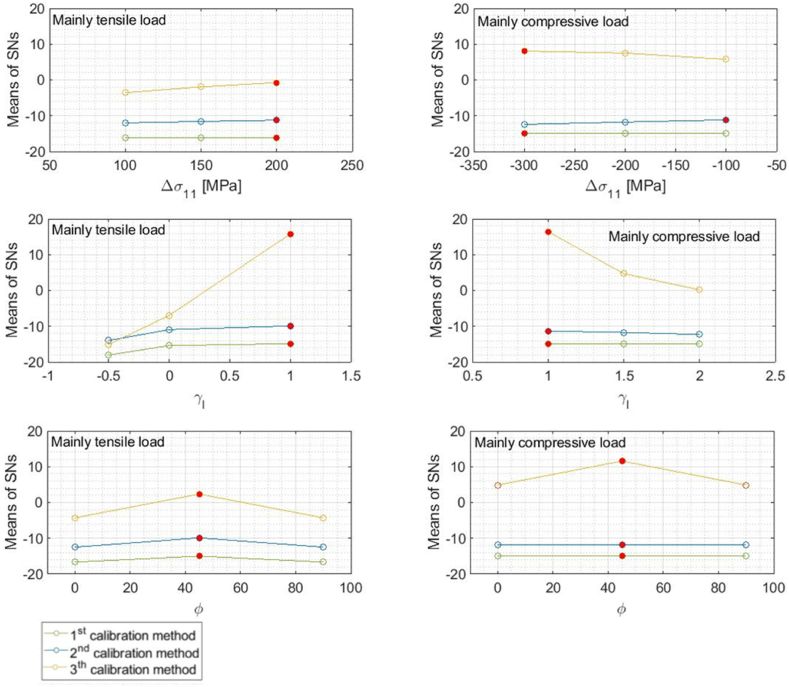
Means of *SN*s of the relative error for each value of the factors *Δσ*_*11,*_*γ*_*l,*_*φ* and each calibration method when the optimal value of *k* is imposed.

The estimate statistical parameters for relative error are reported in Table 8 (tensile case) and Table 9 (compression case). Fig. 9 illustrates the Means of the SNs plotted for each loading characteristic in both considered cases.

**Table 8:** simulation subplan 1. Means and Means of the Standard Deviations of the relative error for each value of the factors *Δσ*_*11.*_*ϒ*_*.*_*φ* and each calibration method when the optimal value of *k* is imposed.

		*calib.1*		*calib.2*		*calib.3*	
		*Mean Error %*	*StDev*	*Mean Error %*	*StDev*	*Mean Error %*	*StDev*
*Δσ*_*11*_*[MPa]*	*−200*	−0.9	6.5	−1.0	4.0	0.1	3.3
	*−150*	−0.9	6.5	−0.9	3.8	0.1	3.0
	*−100*	−0.9	6.4	−0.9	3.6	0.1	2.8
*ϒ*	*1*	−0.7	8.1	−0.6	5.2	0.2	6.4
	*1.5*	−1.0	5.8	−1.0	3.4	4E-02	2.5
	*2*	−1.1	5.4	−1.1	2.9	2E-04	0.2
*φ [°]*	*0*	−1.1	6.9	−1.1	4.2	0.1	3.9
	*45*	−0.6	5.6	−0.6	3.0	1E-02	1.3
	*90*	−1.1	6.9	−1.1	4.2	0.1	3.9

**Table 9:** simulation subplan 2. Means and Means of the Standard Deviations of the relative error for each value of the factors *Δσ*_*11.*_*ϒ*_*.*_*φ* and each calibration method when the optimal value of *k* is imposed.

		*calib.1*		*calib.2*		*calib.3*	
		*Mean Error %*	*StDev Error %*	*Mean Error %*	*StDev Error %*	*Mean Error %*	*StDev Error %*
*Δσ*_*11*_*[MPa]*	*−300*	−1.1	5.5	−1.533	3.9	4E-03	0.7
	*−200*	−1.1	5.5	−1.422	3.6	3E-03	0.6
	*−100*	−1.1	5.5	−1.327	3.4	3E-03	0.6
*ϒ*_*l*_	*1*	−1.1	5.4	−1.3	3.4	2E-04	0.2
	*1.5*	−1.1	5.5	−1.4	3.6	3E-03	0.7
	*2*	−1.1	5.5	−1.5	3.8	8E-03	1.1
*φ [°]*	*0*	−1.1	5.5	−1.4	3.6	5E-03	0.8
	*45*	−1.2	5.4	−1.5	3.6	1E-03	0.3
	*90*	−1.1	5.5	−1.4	3.6	5E-03	0.8

The main variable affecting the measurement is the calibration method. Whenever possible, the best option is always an experimental calibration on the same sample. All three calibration methods exhibit little variation in measurement performance with varying *Δσ*. Generally, an increase in the amplitude of the load implies a higher signal and a reduction of the relative error. However, the direct interaction between *Δσ* and the source of errors leads to an inversion of the trend of *SN* Means in the simulation subplan 2 case for the second calibration method. In this case as well, the results of the *Q/K* ratio are consistent with those of the *SN* ratio; however, the third calibration method exhibits an inversion in the trend of *SN* with respect to the *Q/K* with varying *Δσ*_*11*_ (; detailed results are provided in Appendix 3). In this case, although the average error decreases with the modulus of *Δσ*_*11*_, improving the measurement accuracy, the standard deviation increases faster, resulting in reduced precision.

All calibration methods show an increase in both *SN* and measurement quality in the case of equally biaxial condition. The factor γ affects both the second amplitude and mean load principal components, resulting in a combined effect. The third calibration method proves to be more sensitive to angle variations.

It appears that the measurement performance is improved in the case of monoaxial load tilted of 45°. The optimal angle is a consequence of the relative position respect to the residual stress system which varies between 0 and 90°. Thus, the results demonstrate that the error is minimized when the two systems are aligned.

#### 5.2.3 Deduction and discussion of operational guidelines

The estimate statistical parameter for the relative error are reported first considering the full factorial plan (Table 10) and then considering only the tests with the value of *k* that showed better results from the previous analysis (Table 11).

**Table 10:** Overall relative error statistics.

	*Tensile applied load*				*Compression applied load*			
	*Mean*	*Std.Dev*	*Lower error*	*Higher error*	*Mean*	*Std.Dev*	*Lower error*	*Higher error*
*K*_*0.1*_	6.8	11.7	−28.4	41.9	−6.1	7.0	−27.2	14.9
*K*_*0.2*_	−1.0	4.4	−14.3	12.4	−1.3	3.3	−11.3	8.7
*K*_*0.3*_	0.1	4.9	−14.6	14.8	3E-03	0.7	−2.2	2.2

**Table 11:** Best *k* - relative error statistics.

	*Tensile applied load*				*Compression applied load*			
	*Mean*	*Std.Dev*	*Lower error*	*Higher error*	*Mean*	*Std.Dev*	*Lower error*	*Higher error*
*K*_*0.1*_	−0.9	6.6	−20.8	19.0	−1.1	5.5	−17.5	15.2
*K*_*0.2*_	−0.9	4.1	−13.1	11.3	−1.2	3.1	−10.7	8.2
*K*_*0.3*_	0.1	4.3	−12.9	13.1	2E-03	0.7	−2.1	2.1

The results provide operational directions for TSA application. It is advisable to avoid using a calibration constant evaluated with nominal values for the material characteristics. In this case, considering the overall results and without distinction regarding the direction of the applied load, the error has a mean of about 7 %, varying from −33.2 % to +41.9 %. The measurement performance improves if a null mean load is adopted (*k* = 0), reducing these ranges to −20.8 % to +19 %.

The very low average error should not be misleading. It is the result of a simulation with residual stresses centered around a zero value, so it is much better to refer to the standard deviation and the maximum and minimum error values rather than the average.

For the second calibration method, despite it shows an average error of about 0.1 %, this can range anyway from −14.6 % to +14.8 %. The third calibration method showed a mean error of +0.1 %, with a range from −11.6 % to +8.9 %.

If the best value of *k* is imposed, the performance improvement is less significant than it is in the first method, and the error ranges from −13.1 % to +13.1 % for the second and from −10.7 % to +8.2 % for the second calibration method.

Although the intensity of the applied load does not seem to affect the robustness of the measurement, its direction and orientation are important. Assuming a calibration technique has been chosen and the appropriate value of the k ratio has been set, a compression load with a unit ratio between the two principal components (equibiaxial) is preferred, especially for the third calibration method. Despite the first two methods show a mean error slightly higher, a compression load ensures better precision by providing a lower standard deviation and smaller minimum and maximum errors.

It may appear that using the first and third methods yields a strong difference in performance between the traction and compression cases. However, this discrepancy is due to not symmetrical representation of the load in the two simulation plans. The choice to implement such the plans is related to typical operational conditions end the results reflect these conditions.

## 6 Conclusions and future work

In this study, a statistical approach was employed to investigate the performance of TSA measurements by utilizing an analytical model.

ANOVA and ANOM analyses were conducted to examine the effects of various sources of error on the relationships between the thermal signal and the parameters, as well as their interactions.

The influence of each parameter is closely linked to the ranges selected for the sources of error and for the parameter itself. The study involved the implementation of ranges that reflect real operational conditions, yielding the following results.

• All parameters induce significant variations in the thermal response.
• While the analytical model provides the exact relation between parameters and thermoelastic response, it cannot provide information about their effect on the measured signal, which depends on all noise factors.
• The mean error and the influence of the amplitude of residual stresses are higher in the compression case, characterized by more critical loading conditions and residual stress slightly unbalanced towards the same sign.
• The effect of the residual stress system orientation is lower in the compression case.

The application of Robust Design to the TSA stress measurement system yielded the following results.

• The adoption of a calibration constant estimated with nominal values for the material characteristics implies an error that can range from −28.4 % to +41.9 %, lowering to the interval −20.8 % to +19 % when the mean load effect is compensated by adopting *k* = 0. Thus, the general indication is to prefer an experimental calibration.
• Test conditions that can guarantee the smallest error range include:
  o An experimental calibration with the sample in the same residual stress condition as the component/structure. This implies that the best practice is to use calibration samples extracted from the same component or to calibrate with the use of strain gauges on the component itself.
  o A null mean load (*R* = −1) if the calibration is performed with the first method.
  o A mean load as high as possible if the calibration is performed with the second or third method.
  o A compressive equiaxial load.
• The operative conditions that ensure an error ranging from −2.07 % to +2.07 % involves the calibration with the sample in the same residual stress condition as the component/structure, k = 1.5 and a compressive load.

The proposed work is currently the subject of ongoing research and development. Specifically, experiments have been designed to subject components with varying residual stress states to biaxial loading. To realize a test where the analytical solution for the biaxial stress state is known, the chosen components are Brazilian discs, which will be subjected to compressive loading. Residual stress will be induced plastically deforming the component before discs of the appropriate dimensions are extracted, while their evaluation will be carried out using measurements obtained with a hole-drilling strain gauge for comparison.

## Funding

This research was funded by PRIN: PROGETTI DI RICERCA DI RILEVANTE INTERESSE NAZIONALE – Bando 2022 PNRR: circuLar economy-Oriented DEsign using hybrid-dissimilar joints and sustainable materials for lightweight structures-LODE, grant number P2022MAZHX.

## CRediT authorship contribution statement

**F. Di Carolo:** Writing – review & editing, Writing – original draft, Methodology, Investigation, Formal analysis, Data curation, Conceptualization. **U. Galietti:** Writing – review & editing, Supervision, Methodology, Investigation.

## Declaration of competing interest

The authors declare that they have no known competing financial interests or personal relationships that could have appeared to influence the work reported in this paper.

## Figures and Tables

**Table:** Table A1. 1ANOVA results for simulation subplan 1.

	*Tensile case*							
	*Source*	*Sum Sq.*	*d.f.*	*Mean Sq.*	*F*	*Prob > F*	*Significative (yes/no)*	Note (a.b)**
*1*	**α**	5.7E+06	1	5.7E+06	5.3E+05	0.0E+00	Yes	a
*2*	**ρ**	6.9E+05	1	6.9E+05	6.4E+04	0.0E+00	Yes	a
*3*	**C**_**ε**_	9.0E+06	1	9.0E+06	8.3E+05	0.0E+00	Yes	a
*4*	**E**	1.4E+05	1	1.4E+05	1.3E+04	0.0E+00	Yes	a
*5*	**∂E/∂T**	4.0E+04	1	4.0E+04	3.7E+03	0.0E+00	Yes	a
*6*	**σ**_**r11**_	5.6E+06	6	9.3E+05	8.6E+04	0.0E+00	Yes	a
*7*	**Δσ**_**rp**_	4.0E+05	2	2.0E+05	1.8E+04	0.0E+00	Yes	a
*8*	**θ**	4.2E+04	2	2.1E+04	1.9E+03	0.0E+00	Yes	a
*9*	α *ρ	5.0E+02	1	5.0E+02	4.6E+01	1.2E-11	Yes	a
*10*	α * C_**ε**_	6.5E+03	1	6.5E+03	6.0E+02	2.1E-132	Yes	a
*11*	α *E	2.6E-01	1	2.6E-01	2.4E-02	8.8E-01	No	a
*12*	α *∂E/∂T	2.3E-04	1	2.3E-04	2.1E-05	1.0E+00	No	a
*13*	α *sr11	8.9E-01	6	1.5E-01	1.4E-02	1.0E+00	No	a
*14*	α * Δσ_**rp**_	2.2E-01	2	1.1E-01	9.9E-03	9.9E-01	No	a
*15*	α *θ	1.3E-01	2	6.5E-02	6.0E-03	9.9E-01	No	a
*16*	ρ * C_**ε**_	7.7E+02	1	7.7E+02	7.1E+01	3.0E-17	Yes	a
*17*	ρ *E	9.7E+00	1	9.7E+00	8.9E-01	3.4E-01	No	b
*18*	ρ *∂E/∂T	2.4E+00	1	2.4E+00	2.2E-01	6.4E-01	No	b
*19*	ρ * σ_**r11**_	4.8E+02	6	8.0E+01	7.3E+00	7.4E-08	Yes	a
*20*	ρ * Δσ_**rp**_	3.1E+01	2	1.5E+01	1.4E+00	2.4E-01	No	b
*21*	ρ *θ	2.5E+00	2	1.3E+00	1.2E-01	8.9E-01	No	b
*22*	**C**_ε_*E	1.6E+02	1	1.6E+02	1.5E+01	1.2E-04	Yes	a
*23*	**C**_ε_*∂E/∂T	4.8E+01	1	4.8E+01	4.5E+00	3.5E-02	No	b
*24*	**C**_ε_* σ_**r11**_	6.3E+03	6	1.0E+03	9.6E+01	3.1E-121	Yes	a
*25*	**C**_ε_* Δσ_**rp**_	4.2E+02	2	2.1E+02	2.0E+01	3.1E-09	Yes	a
*26*	**C**_ε_*θ	5.0E+01	2	2.5E+01	2.3E+00	1.0E-01	No	b
*27*	E*∂E/∂T	2.5E+02	1	2.5E+02	2.3E+01	1.3E-06	Yes	a
*28*	E* σ_**r11**_	3.4E+04	6	5.7E+03	5.2E+02	0.0E+00	Yes	a
*29*	E* Δσ_**rp**_	2.4E+03	2	1.2E+03	1.1E+02	1.4E-48	Yes	a
*30*	E* θ	2.6E+02	2	1.3E+02	1.2E+01	6.4E-06	Yes	a
*31*	∂E/∂T * σ_**r11**_	9.6E+03	6	1.6E+03	1.5E+02	6.8E-188	Yes	a
*32*	∂E/∂T * Δσ_**rp**_	6.7E+02	2	3.4E+02	3.1E+01	3.3E-14	Yes	a
*33*	∂E/∂T *θ	7.5E+01	2	3.7E+01	3.4E+00	3.2E-02	No	b
*34*	**σ**_r11_* Δσ_**rp**_	9.6E-01	12	8.0E-02	7.4E-03	1.0E+00	No	b
*35*	**σ**_r11_*θ	1.0E+00	12	8.5E-02	7.9E-03	1.0E+00	No	b
*36*	**Δσ**_rp_*θ	2.3E+04	4	5.8E+03	5.4E+02	0.0E+00	Yes	a
*37*	α *ρ * C_**ε**_	6.7E-01	1	6.7E-01	6.2E-02	8.0E-01	No	b
*38*	α *ρ *E	1.4E-02	1	1.4E-02	1.3E-03	9.7E-01	No	a
*39*	α *ρ *∂E/∂T	4.1E-02	1	4.1E-02	3.8E-03	9.5E-01	No	a
*40*	α *ρ * σ_**r11**_	1.5E+00	6	2.5E-01	2.3E-02	1.0E+00	No	a
*41*	α *ρ * Δσ_**rp**_	9.3E-01	2	4.7E-01	4.3E-02	9.6E-01	No	a
*42*	α *ρ *θ	4.4E-02	2	2.2E-02	2.0E-03	1.0E+00	No	a
*43*	α * C_ε_*E	7.1E-02	1	7.1E-02	6.6E-03	9.4E-01	No	a
*44*	α * C_ε_*∂E/∂T	9.5E-02	1	9.5E-02	8.8E-03	9.3E-01	No	a
*45*	α * C_ε_* σ_**r11**_	7.8E-01	6	1.3E-01	1.2E-02	1.0E+00	No	a
*46*	α * C_ε_* Δσ_**rp**_	4.0E-02	2	2.0E-02	1.8E-03	1.0E+00	No	a
*47*	α * C_ε_*θ	8.5E-03	2	4.3E-03	3.9E-04	1.0E+00	No	a
*48*	α *E*∂E/∂T	2.9E-02	1	2.9E-02	2.7E-03	9.6E-01	No	a
*49*	α *E* σ_**r11**_	4.5E-01	6	7.5E-02	6.9E-03	1.0E+00	No	a
*50*	α *E* Δσ_**rp**_	2.4E-02	2	1.2E-02	1.1E-03	1.0E+00	No	a
*51*	α *E* θ	6.8E-01	2	3.4E-01	3.1E-02	9.7E-01	No	a
*52*	α *∂E/∂T * σ_**r11**_	6.7E-01	6	1.1E-01	1.0E-02	1.0E+00	No	a
*53*	α *∂E/∂T * Δσ_**rp**_	2.8E-02	2	1.4E-02	1.3E-03	1.0E+00	No	a
*54*	α *∂E/∂T *θ	1.6E-02	2	8.1E-03	7.5E-04	1.0E+00	No	a
*55*	α *sr11* Δσ_**rp**_	6.8E-01	12	5.7E-02	5.2E-03	1.0E+00	No	a
*56*	α * σ_r11_*θ	7.6E-01	12	6.3E-02	5.8E-03	1.0E+00	No	a
*57*	α * Δσ_rp_* θ	1.7E-01	4	4.2E-02	3.9E-03	1.0E+00	No	a
*58*	ρ * C_ε_*E	1.4E-02	1	1.4E-02	1.2E-03	9.7E-01	No	b
*59*	ρ * C_ε_*∂E/∂T	1.2E-01	1	1.2E-01	1.1E-02	9.2E-01	No	b
*60*	ρ * C_ε_* σ_**r11**_	1.2E+00	6	2.1E-01	1.9E-02	1.0E+00	No	b
*61*	ρ * C_ε_* Δσ_**rp**_	4.7E-01	2	2.4E-01	2.2E-02	9.8E-01	No	b
*62*	ρ * C_ε_*θ	2.8E-02	2	1.4E-02	1.3E-03	1.0E+00	No	b
*63*	ρ *E*∂E/∂T	1.2E-01	1	1.2E-01	1.1E-02	9.2E-01	No	b
*64*	ρ *E* σ_**r11**_	1.8E+00	6	3.0E-01	2.8E-02	1.0E+00	No	b
*65*	ρ *E* Δσ_**rp**_	2.7E-01	2	1.4E-01	1.2E-02	9.9E-01	No	b
*66*	ρ *E* θ	2.0E-02	2	1.0E-02	9.4E-04	1.0E+00	No	b
*67*	ρ *∂E/∂T * σ_**r11**_	6.8E-01	6	1.1E-01	1.1E-02	1.0E+00	No	b
*68*	ρ *∂E/∂T * Δσ_**rp**_	8.9E-02	2	4.4E-02	4.1E-03	1.0E+00	No	b
*69*	ρ *∂E/∂T *θ	1.6E-01	2	7.8E-02	7.2E-03	9.9E-01	No	b
*70*	ρ * σ_r11_* Δσ_**rp**_	1.9E+00	12	1.6E-01	1.4E-02	1.0E+00	No	b
*71*	ρ * σ_r11_*θ	1.6E+00	12	1.3E-01	1.2E-02	1.0E+00	No	b
*72*	ρ * Δσ_rp_*θ	1.8E+00	4	4.5E-01	4.2E-02	1.0E+00	No	b
*73*	**C**_ε_*E*∂E/∂T	1.5E-01	1	1.5E-01	1.4E-02	9.1E-01	No	b
*74*	**C**_ε_*E* σ_**r11**_	4.4E+01	6	7.3E+00	6.8E-01	6.7E-01	No	b
*75*	**C**_ε_*E* Δσ_**rp**_	1.9E+00	2	9.4E-01	8.6E-02	9.2E-01	No	b
*76*	**C**_ε_*E* θ	1.5E-01	2	7.3E-02	6.8E-03	9.9E-01	No	b
*77*	**C**_ε_*∂E/∂T * σ_**r11**_	1.0E+01	6	1.7E+00	1.6E-01	9.9E-01	No	b
*78*	**C**_ε_*∂E/∂T * Δσ_**rp**_	1.5E+00	2	7.3E-01	6.7E-02	9.3E-01	No	b
*79*	**C**_ε_*∂E/∂T *θ	2.1E-01	2	1.0E-01	9.6E-03	9.9E-01	No	b
*80*	**C**_ε_* σ_r11_* Δσ_**rp**_	6.8E-01	12	5.7E-02	5.2E-03	1.0E+00	No	b
*81*	**C**_ε_* σ_r11_*θ	2.0E+00	12	1.7E-01	1.5E-02	1.0E+00	No	b
*82*	**C**_ε_* Δσ_rp_*θ	2.4E+01	4	6.0E+00	5.5E-01	7.0E-01	No	b
*83*	E*∂E/∂T * σ_**r11**_	5.6E+01	6	9.3E+00	8.6E-01	5.3E-01	No	b
*84*	E*∂E/∂T * Δσ_**rp**_	1.3E+00	2	6.7E-01	6.2E-02	9.4E-01	No	b
*85*	E*∂E/∂T *θ	3.3E-01	2	1.6E-01	1.5E-02	9.9E-01	No	b
*86*	E* σ_r11_* Δσ_**rp**_	1.3E+00	12	1.1E-01	9.9E-03	1.0E+00	No	b
*87*	E* σ_r11_*θ	1.1E+00	12	8.8E-02	8.1E-03	1.0E+00	No	b
*88*	E* Δσ_rp_*θ	1.5E+02	4	3.8E+01	3.5E+00	7.3E-03	Yes	a
*89*	∂E/∂T * σ_r11_* Δσ_**rp**_	2.8E+00	12	2.4E-01	2.2E-02	1.0E+00	No	b
*90*	∂E/∂T * σ_r11_*θ	1.1E+00	12	9.3E-02	8.6E-03	1.0E+00	No	b
*91*	∂E/∂T * Δσ_rp_*θ	3.9E+01	4	9.9E+00	9.1E-01	4.6E-01	No	b
*92*	**σ**_r11_* Δσ_rp_*θ	2.0E+00	24	8.4E-02	7.7E-03	1.0E+00	No	b
	**Error**	8.9E+06	816102	1.1E+01				
	**Total**	3.1E+07	816479					

**a as expected from the analytical model. *b* should be significative from the analytical model. but covered by noise.

**Table A1. 2:** ANOVA results for simulation subplan 2.

	*Compression case*							
	*Source*	*Sum Sq.*	*d.f.*	*Mean Sq.*	*F*	*Prob > F*	*Significative (yes/no)*	Note (a.b)**
*1*	**α**	7.2E+06	1	7.2E+06	1.6E+07	0.0E+00	Yes	a
*2*	**ρ**	6.9E+05	1	6.9E+05	1.5E+06	0.0E+00	Yes	a
*3*	**C**_**ε**_	9.0E+06	1	9.0E+06	1.9E+07	0.0E+00	Yes	a
*4*	**E**	2.1E+05	1	2.1E+05	4.6E+05	0.0E+00	Yes	a
*5*	**∂E/∂T**	5.9E+04	1	5.9E+04	1.3E+05	0.0E+00	Yes	a
*6*	**σ**_**r11**_	7.0E+06	6	1.2E+06	2.5E+06	0.0E+00	Yes	a
*7*	**Δσ**_**rp**_	7.1E+05	2	3.5E+05	7.7E+05	0.0E+00	Yes	a
*8*	**θ**	9.4E+02	2	4.7E+02	1.0E+03	0.0E+00	Yes	a
*9*	α *ρ	6.3E+02	1	6.3E+02	1.4E+03	3.8E-297	Yes	a
*10*	α * C_**ε**_	8.1E+03	1	8.1E+03	1.8E+04	0.0E+00	Yes	a
*11*	α *E	4.9E-06	1	4.9E-06	1.1E-05	1.0E+00	No	a
*12*	α *∂E/∂T	4.5E-03	1	4.5E-03	9.7E-03	9.2E-01	No	a
*13*	α *sr11	5.5E-02	6	9.2E-03	2.0E-02	1.0E+00	No	a
*14*	α * Δσ_**rp**_	8.4E-04	2	4.2E-04	9.1E-04	1.0E+00	No	a
*15*	α *θ	1.0E-01	2	5.1E-02	1.1E-01	9.0E-01	No	a
*16*	ρ * C_**ε**_	7.7E+02	1	7.7E+02	1.7E+03	0.0E+00	Yes	a
*17*	ρ *E	1.8E+01	1	1.8E+01	4.0E+01	2.8E-10	Yes	a
*18*	ρ *∂E/∂T	5.4E+00	1	5.4E+00	1.2E+01	6.2E-04	Yes	a
*19*	ρ * σ_**r11**_	6.1E+02	6	1.0E+02	2.2E+02	3.1E-281	Yes	a
*20*	ρ * Δσ_**rp**_	5.8E+01	2	2.9E+01	6.3E+01	3.3E-28	Yes	a
*21*	ρ *θ	4.8E-02	2	2.4E-02	5.2E-02	9.5E-01	No	b
*22*	**C**_ε_*E	2.4E+02	1	2.4E+02	5.1E+02	4.9E-113	Yes	a
*23*	**C**_ε_*∂E/∂T	6.7E+01	1	6.7E+01	1.5E+02	1.4E-33	Yes	a
*24*	**C**_ε_* σ_**r11**_	7.9E+03	6	1.3E+03	2.9E+03	0.0E+00	Yes	a
*25*	**C**_ε_* Δσ_**rp**_	8.0E+02	2	4.0E+02	8.7E+02	0.0E+00	Yes	a
*26*	**C**_ε_*θ	1.2E+00	2	5.8E-01	1.2E+00	2.9E-01	No	b
*27*	E*∂E/∂T	3.7E+02	1	3.7E+02	8.0E+02	5.5E-175	Yes	a
*28*	E* σ_**r11**_	4.3E+04	6	7.2E+03	1.6E+04	0.0E+00	Yes	a
*29*	E* Δσ_**rp**_	4.4E+03	2	2.2E+03	4.7E+03	0.0E+00	Yes	a
*30*	E* θ	6.3E+00	2	3.1E+00	6.8E+00	1.1E-03	Yes	a
*31*	∂E/∂T * σ_**r11**_	1.2E+04	6	2.0E+03	4.4E+03	0.0E+00	Yes	a
*32*	∂E/∂T * Δσ_**rp**_	1.2E+03	2	6.2E+02	1.3E+03	0.0E+00	Yes	a
*33*	∂E/∂T *θ	1.7E+00	2	8.7E-01	1.9E+00	1.5E-01	No	b
*34*	**σ**_r11_* Δσ_**rp**_	1.4E-01	12	1.2E-02	2.5E-02	1.0E+00	No	b
*35*	**σ**_r11_*θ	1.4E-01	12	1.2E-02	2.5E-02	1.0E+00	No	b
*36*	**Δσ**_rp_*θ	5.3E+02	4	1.3E+02	2.9E+02	5.4E-249	Yes	a
*37*	α *ρ * C_**ε**_	6.5E-01	1	6.5E-01	1.4E+00	2.4E-01	No	b
*38*	α *ρ *E	8.8E-04	1	8.8E-04	1.9E-03	9.7E-01	No	a
*39*	α *ρ *∂E/∂T	4.0E-03	1	4.0E-03	8.6E-03	9.3E-01	No	a
*40*	α *ρ * σ_**r11**_	8.0E-02	6	1.3E-02	2.9E-02	1.0E+00	No	a
*41*	α *ρ * Δσ_**rp**_	7.1E-03	2	3.6E-03	7.7E-03	9.9E-01	No	a
*42*	α *ρ *θ	2.8E-02	2	1.4E-02	3.0E-02	9.7E-01	No	a
*43*	α * C_ε_*E	3.4E-03	1	3.4E-03	7.3E-03	9.3E-01	No	a
*44*	α * C_ε_*∂E/∂T	6.4E-03	1	6.4E-03	1.4E-02	9.1E-01	No	a
*45*	α * C_ε_* σ_**r11**_	1.0E-01	6	1.7E-02	3.8E-02	1.0E+00	No	a
*46*	α * C_ε_* Δσ_**rp**_	1.3E-02	2	6.7E-03	1.4E-02	9.9E-01	No	a
*47*	α * C_ε_*θ	1.1E-02	2	5.5E-03	1.2E-02	9.9E-01	No	a
*48*	α *E*∂E/∂T	4.7E-02	1	4.7E-02	1.0E-01	7.5E-01	No	a
*49*	α *E* σ_**r11**_	1.4E-02	6	2.3E-03	5.0E-03	1.0E+00	No	a
*50*	α *E* Δσ_**rp**_	4.5E-02	2	2.2E-02	4.9E-02	9.5E-01	No	a
*51*	α *E* θ	1.1E-02	2	5.4E-03	1.2E-02	9.9E-01	No	a
*52*	α *∂E/∂T * σ_**r11**_	6.4E-02	6	1.1E-02	2.3E-02	1.0E+00	No	a
*53*	α *∂E/∂T * Δσ_**rp**_	6.0E-03	2	3.0E-03	6.5E-03	9.9E-01	No	a
*54*	α *∂E/∂T *θ	1.9E-02	2	9.4E-03	2.0E-02	9.8E-01	No	a
*55*	α *sr11* Δσ_**rp**_	1.8E-01	12	1.5E-02	3.3E-02	1.0E+00	No	a
*56*	α * σ_r11_*θ	1.1E-01	12	9.0E-03	1.9E-02	1.0E+00	No	a
*57*	α * Δσ_rp_* θ	1.2E-01	4	3.0E-02	6.6E-02	9.9E-01	No	a
*58*	ρ * C_ε_*E	7.8E-03	1	7.8E-03	1.7E-02	9.0E-01	No	b
*59*	ρ * C_ε_*∂E/∂T	1.7E-04	1	1.7E-04	3.6E-04	9.8E-01	No	b
*60*	ρ * C_ε_* σ_**r11**_	9.9E-01	6	1.6E-01	3.6E-01	9.1E-01	No	b
*61*	ρ * C_ε_* Δσ_**rp**_	7.1E-02	2	3.5E-02	7.6E-02	9.3E-01	No	b
*62*	ρ * C_ε_*θ	1.8E-02	2	9.0E-03	2.0E-02	9.8E-01	No	b
*63*	ρ *E*∂E/∂T	5.1E-02	1	5.1E-02	1.1E-01	7.4E-01	No	b
*64*	ρ *E* σ_**r11**_	3.2E+00	6	5.3E-01	1.2E+00	3.3E-01	No	b
*65*	ρ *E* Δσ_**rp**_	2.3E-01	2	1.1E-01	2.4E-01	7.8E-01	No	b
*66*	ρ *E* θ	1.0E-05	2	5.1E-06	1.1E-05	1.0E+00	No	b
*67*	ρ *∂E/∂T * σ_**r11**_	1.2E+00	6	2.1E-01	4.5E-01	8.5E-01	No	b
*68*	ρ *∂E/∂T * Δσ_**rp**_	1.0E-01	2	5.2E-02	1.1E-01	8.9E-01	No	b
*69*	ρ *∂E/∂T *θ	2.5E-02	2	1.2E-02	2.7E-02	9.7E-01	No	b
*70*	ρ * σ_r11_* Δσ_**rp**_	1.7E-01	12	1.4E-02	3.1E-02	1.0E+00	No	b
*71*	ρ * σ_r11_*θ	2.0E-01	12	1.6E-02	3.5E-02	1.0E+00	No	b
*72*	ρ * Δσ_rp_*θ	1.2E-01	4	2.9E-02	6.3E-02	9.9E-01	No	b
*73*	**C**_ε_*E*∂E/∂T	5.3E-01	1	5.3E-01	1.1E+00	2.8E-01	No	b
*74*	**C**_ε_*E* σ_**r11**_	4.8E+01	6	8.0E+00	1.7E+01	3.0E-20	Yes	a
*75*	**C**_ε_*E* Δσ_**rp**_	4.5E+00	2	2.3E+00	4.9E+00	7.5E-03	Yes	a
*76*	**C**_ε_*E* θ	2.0E-02	2	9.9E-03	2.1E-02	9.8E-01	No	b
*77*	**C**_ε_*∂E/∂T * σ_**r11**_	1.4E+01	6	2.4E+00	5.2E+00	2.1E-05	Yes	a
*78*	**C**_ε_*∂E/∂T * Δσ_**rp**_	1.5E+00	2	7.5E-01	1.6E+00	2.0E-01	No	b
*79*	**C**_ε_*∂E/∂T *θ	2.5E-02	2	1.3E-02	2.7E-02	9.7E-01	No	b
*80*	**C**_ε_* σ_r11_* Δσ_**rp**_	1.2E-01	12	1.0E-02	2.2E-02	1.0E+00	No	b
*81*	**C**_ε_* σ_r11_*θ	2.0E-01	12	1.6E-02	3.5E-02	1.0E+00	No	b
*82*	**C**_ε_* Δσ_rp_*θ	5.7E-01	4	1.4E-01	3.1E-01	8.7E-01	No	b
*83*	E*∂E/∂T * σ_**r11**_	7.6E+01	6	1.3E+01	2.7E+01	8.3E-33	Yes	a
*84*	E*∂E/∂T * Δσ_**rp**_	7.3E+00	2	3.7E+00	7.9E+00	3.6E-04	Yes	a
*85*	E*∂E/∂T *θ	3.9E-02	2	1.9E-02	4.2E-02	9.6E-01	No	b
*86*	E* σ_r11_* Δσ_**rp**_	9.3E-02	12	7.7E-03	1.7E-02	1.0E+00	No	b
*87*	E* σ_r11_*θ	1.7E-01	12	1.4E-02	3.1E-02	1.0E+00	No	b
*88*	E* Δσ_rp_*θ	3.2E+00	4	8.0E-01	1.7E+00	1.4E-01	No	b
*89*	∂E/∂T * σ_r11_* Δσ_**rp**_	1.9E-01	12	1.6E-02	3.4E-02	1.0E+00	No	b
*90*	∂E/∂T * σ_r11_*θ	5.9E-02	12	4.9E-03	1.1E-02	1.0E+00	No	b
*91*	∂E/∂T * Δσ_rp_*θ	5.8E-01	4	1.5E-01	3.2E-01	8.7E-01	No	b
*92*	**σ**_r11_* Δσ_rp_*θ	4.7E-01	24	1.9E-02	4.2E-02	1.0E+00	No	b
	**Error**	3.8E+05	816102	4.6E-01				
	**Total**	2.5E+07	816479					

**a as expected from the analytical model. *b* should be significative from the analytical model. but covered by noise.

**Fig. A2.1:**
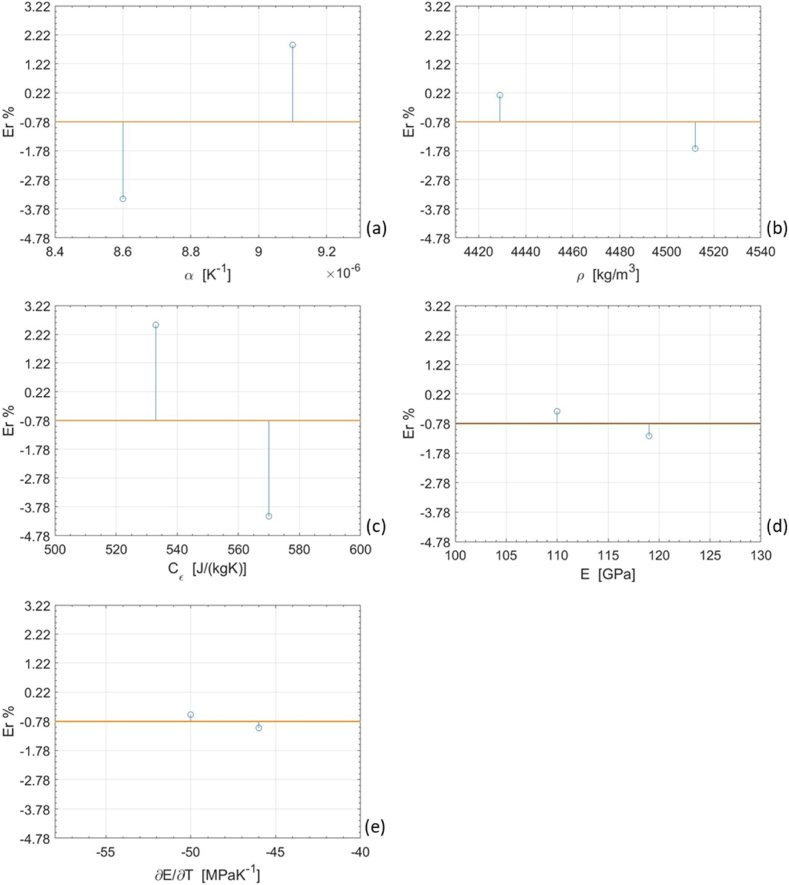
ANOM results for the simulation subplan 1. The Means are plotted for the different levels of the material characteristics (a) *α*, (b) ρ, (c) *C*_*ε*_, (d) *E* and (e) *∂E\∂T.*

**Fig. A2.2:**
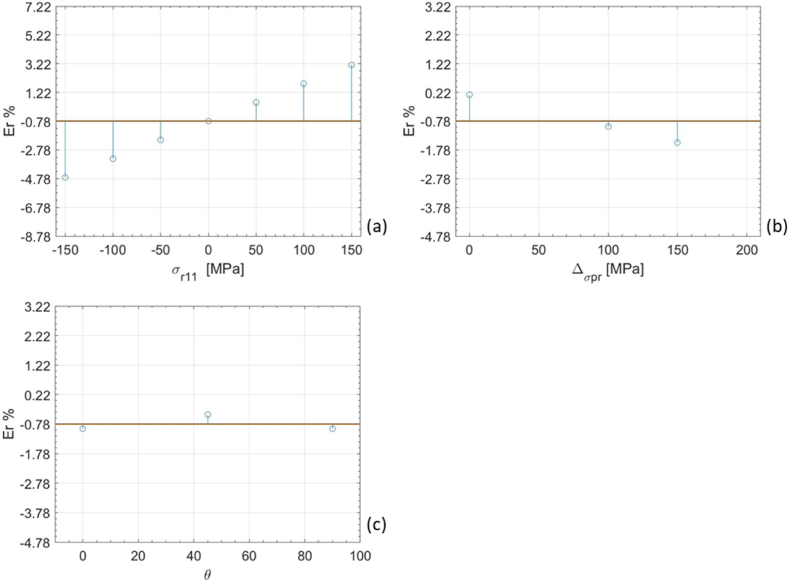
ANOM results for the simulation subplan 1. The Means (blue circles) are plotted for the different levels of the residual stress characteristics (a) *σ*_*r11*_, (b) Δσ_rp_ and (c) *θ.*

**Fig. A2.3:**
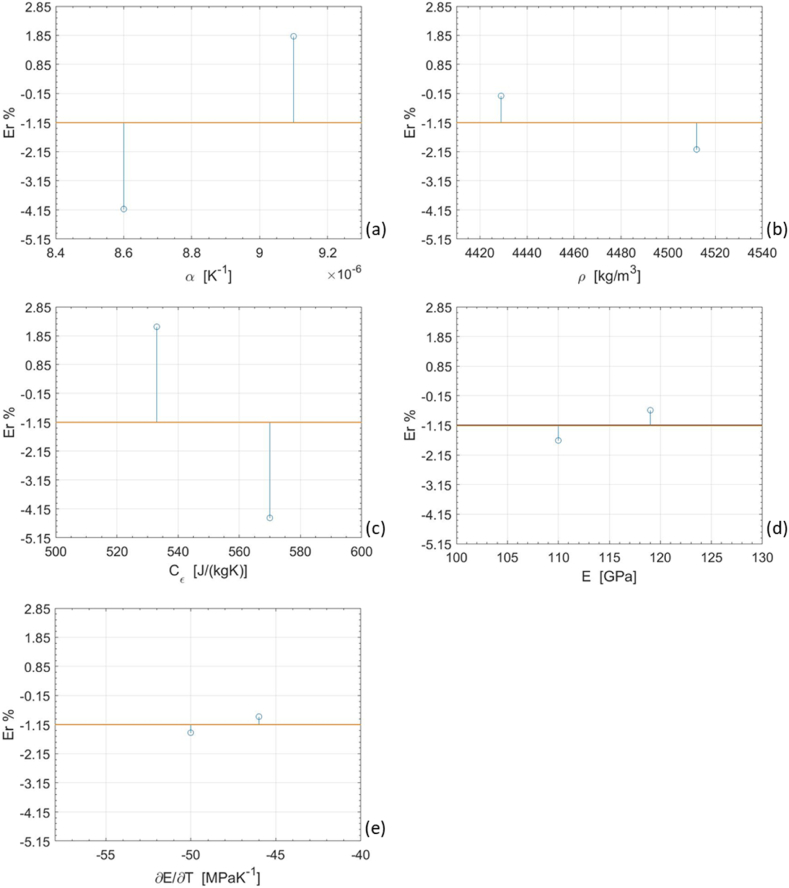
ANOM results for simulation subplan 2. The Means (blue circles) are plotted for the different levels of the material characteristics (a) *α*, (b) ρ, (c) *C*_*ε*_, (d) *E* and (e) *∂E\∂T.*

**Fig. A2.4:**
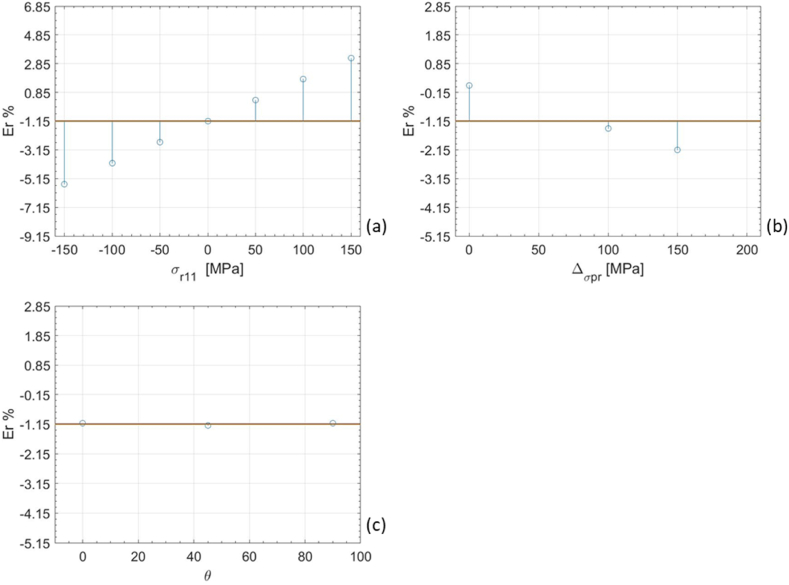
ANOM results the simulation subplan 2. The Means (blue circles) re plotted for the different levels of the residual stress characteristics (a) *σ*_*r11*_, (b) Δσ_rp_ and (c) *θ.*

**Fig. A3.1:**
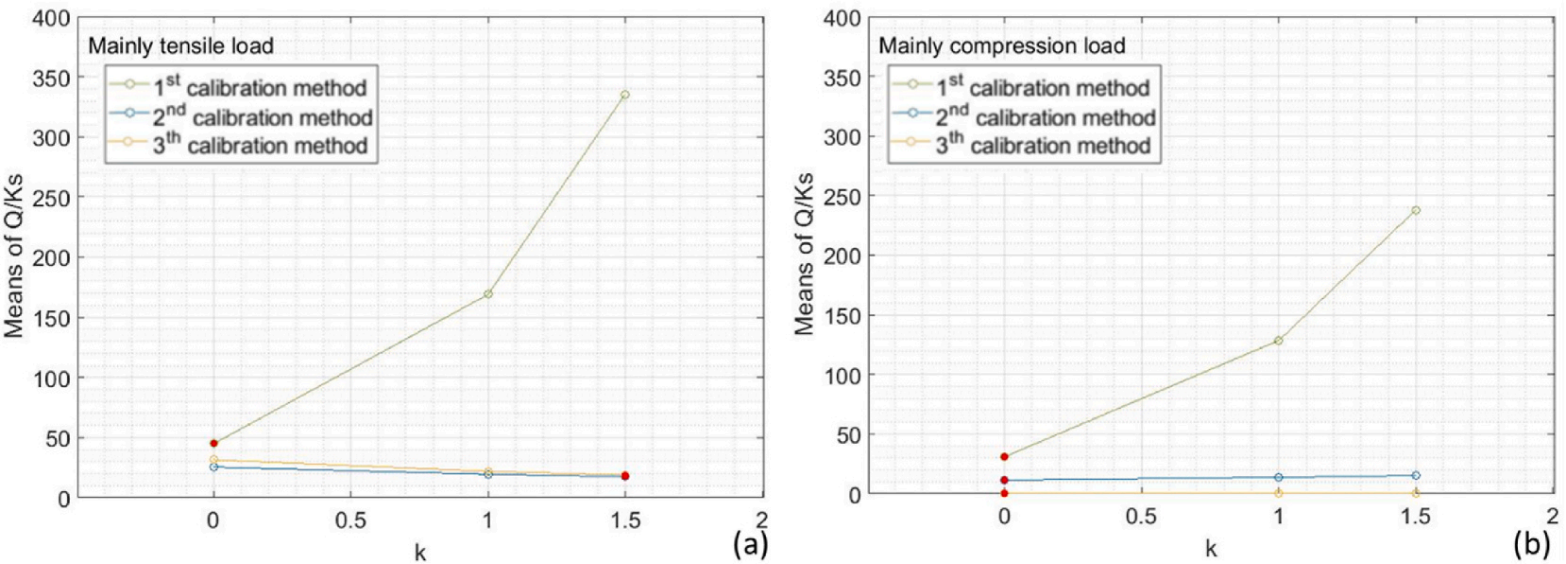
Means of Q/Ks of the relative error for each value of the control factor k and each calibration method for (a) the simulation subplan 1 (b) the simulation subplan 2.

**Fig. A3.2:**
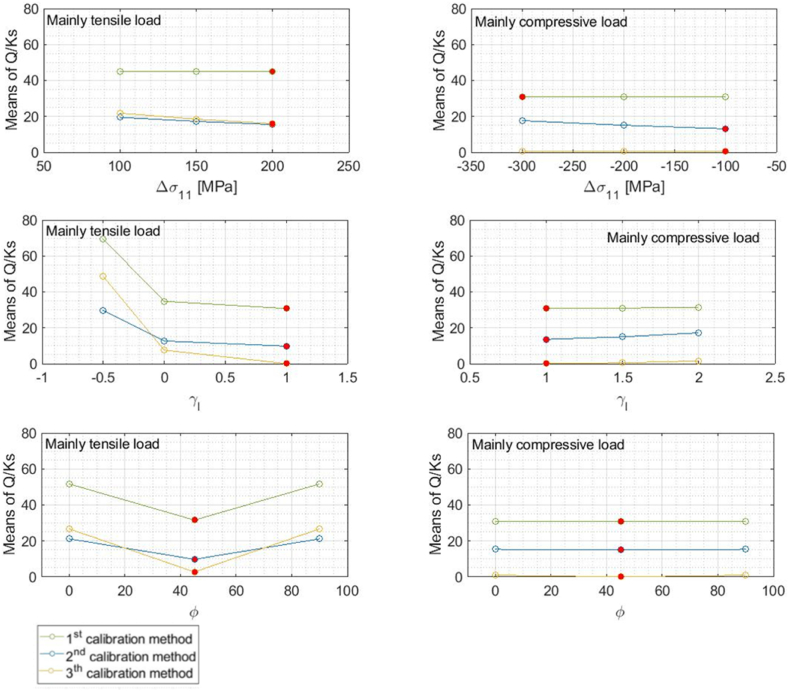
Means of *Q/K*s of the relative error for each value of the the factors *Δσ*_*11,*_*γ*_*l,*_*φ* and each calibration method when the optimal value of *k* is imposed.

## APPENDIX 1 : ANOVA Tables

Common influential two-factor interactions across both loading cases are labelled as numbers *9, 10, 16, 19, 22, 24, 25, 27, 28, 29, 30, 31, 32, 36* in the 1st column of Table A1.1 and Table A1.2. Additionally, two-factor interactions numbered *17, 18, 20* and *23* are significant solely in the compression case. This could be attributed to the parameter *ρ* having a relatively smaller range of variation compared to other parameters, thereby potentially influencing its interaction with parameters leading to a reduction in the amplitude of the thermal signal. This significance is more pronounced in the second simulation plan, where the sum of the principal stress amplitude ranges from −200 to −900 MPa, compared to the first plan where it varies from 50 to 600 MPa.
